# Hints upon the Profession of Medicine

**Published:** 1851-10

**Authors:** Wm. Maxwell Wood

**Affiliations:** Surgeon U. S. Navy


### BUFFALO MEDICAL JOURNAL

AND

### MONTHLY REVIEW.

VOL. 7. OCTOBER, 1851. NO. 5.

### ORIGINAL COMMUNICATIONS.

ART. I. — *Hints upon the Profession of Medicine.* By WM. MAXWELL WOOD, M. D., Surgeon U. S. Navy.

(Continued from Page 203.)

Familiar as the student of medicine is with such and similar transactions, he may well be excused for paying more deference to the calm, cautious, and philosophic decisions of his profession, than to visionary schemes and fanciful systems, however supported by the authority of names, rank, and numbers.

Whilst showing the unavoidable obligations which the profession of medicine has to the pursuit of truth, and the varied means by which it pursues the investigation, it cannot be supposed, by any, that it is claimed for every individual practicing the healing art, even under the sanction of a college certificate, that he represents that profession; — far from it. Unfortunately the popular judgment of the profession is generally formed from those members who misrepresent it; and the illiberal and ungentlemanly intercourse which grows out of professional rivalry, has given rise to a proverb, which is passed from mouth to mouth, conveying a fallacy in reference to the profession at large. We shall endeavor to disabuse the public mind of that fallacy.

"Doctors differ," is the general expression of the general sentiment, and if it is meant that like rival statesmen, rival authors, rival mechanics, they manifest the jealousies, and business animosities of human nature, there is no disputing the unhappy fact; indeed, it may be admitted that their differences, or business quarrels are more conspicuous, perhaps more frequent, because, in the pursuit of their vocation they are brought into personal contact with each other. But the popular interpretation of the proverb "Doctors differ," means much more than business quarrels. It means, that each individual of the profession, is the embodiment of certain professional principles, which differ from those held by each other individual, and that the difference is a legitimate one. It implies the fallacy that each practitioner is the founder, or inventor of his own peculiar system of practice. From the nature of the view we have taken of the profession of medicine, it must be seen that they cannot be so, but, in proportion as the practitioners of medicine are instructed in the scientific truths and principles of their profession, they must agree. They represent general truths, not individual opinions. Information, with honesty, must produce concord. But if illy instructed, each practitioner, instead of being influenced by fixed principles, may become the advocate of ignorant and opposite opinions. For instance: a case of disease may be marked by known and determined symptoms, and for these symptoms or their cause, there may be a fixed and rational mode of treatment. Now, gather around this case practitioners who are badly informed, ignorant of the mode of investigating disease, ignorant of the signification of symptoms, and ignorant of their management when ascertained, and each one will make his own guess, and maintain it with a warmth proportioned to its want of truth. Bring to the same case well informed practitioners from America, from England, from Germany, from France, and they will agree as to the nature of the disease, and as to the principles of its treatment.

Differences, therefore, among medical men, however disgraceful to the individuals, cannot justly be imputed to the profession of medicine. From these circumstances it is evident that none are worthy of confidence, as practitioners of medicine, but those who, with intellectual capacity for acquirement, have had mental training, time and opportunity for studying the vast range of medical science, and that industry, application, and sense of moral obligation which ensure the fulfilment of the high responsibilities of the profession of medicine. Such a rule of judgment would exclude a great many who are now successful practitioners, but if people would only keep before them the common sense view of the science of medicine, and see it not a string of mystery and magic, but one requiring, for even the most talented, long and laborious application, they would exercise a safer discrimination as to the qualifications of their medical attendants. There are undoubtedly many practitioners who honestly believe themselves qualified for the duties they undertake, simply because they have not penetrated sufficiently far within the domain of medical knowledge to perceive its broad extent and varied character, or else are naturally incapable of this extended vision. Such ignorance may give a boldness in proffering relief, which better information would very much moderate.

Besides those too credulous persons who believe in all medical novelties and pretensions, there are others who repeat the maxim " Nature is the best physician," and refuse all aid for their maladies. Such sceptics imagine that their view is sustained, because the most intelligent teachers of medical science inculcate that our wisdom is to be derived from the observation of the laws of nature. There is no relation, whatever, between the popular opinion to which we have alluded, and scientific obedience to the laws of nature. An acquaintance with these laws implies a vast amount of information, and teaches the natural means by which their irregularities and aberrations are to be corrected; and teaches also the certain and overwhelming danger, which permits the aberrations of nature's laws to go without correction. If an individual had one of his arteries divided, and saw his life blood pouring forth with fearful rapidity, he would not wait and say " nature is the best physician," but he would apply to some one who, from the observation of the processes of nature, has learned how to stop the flow of blood. As great and as fatal injuries, from the progress of disease, may be going on in internal organs; the truster in nature, or rather, the wilful contemner of nature does not see it, and therefore does nothing and dies of a " medicable wound," which would be very apparent to the physician whose eye had been taught to search it out. It is true, nature is the best physician, but she requires an industrious and devoted worship to secure her attendance, and is very apt to visit the penalty of disease and death upon those who neglect her. The best ministers of nature, then, are those who most assiduously study her laws; and an acquaintance with those laws constitutes extensive learning. Unfortunately, too many of those who look to the physician for medical aid, expect him to be the bold controller of nature, instead of her vigilant observer, faithful follower, and intelligent assistant. Certain and mechanical results are confidently looked for where true and high science teach that nothing but probabilities could be reached by human skill. Correct scientific or professional knowledge increases those probabilities, but those who study the science of medicine most profoundly, know, that while their chances of arresting disease, depend upon the extent and variety of their acquirements, nothing attainable by human faculties can give the power of certainty. Hence the conscientious practitioner of medicine is stimulated to add to his professional attainments, and feels it to be a moral duty to go from one acquirement to another.

One so enlightened will promise no certain cure for the slightest ailment, for he well knows that under the mysterious agency of vital laws, which are hidden by Providence from the scrutiny of man, death may result from the slightest derangement of the human organism:—a scratch may terminate in fatal mortification; lock-jaw and death result from the extraction of a tooth, or even a too close clipping of the nails; and the sting of a wasp has been known to terminate life in fifteen minutes. Hence the well informed physician, although he may be confident of his abilities to select with knowledge and judgment among the various remedies with which he may be familiar, and though he may know that they are naturally suited to the case he may be treating, awaits with cautious hope, rather than bold assurance, a result it is not with him to determine.

The uncertainty which attends the profession of medicine, is applicable to every avocation connected with the laws of nature. All such professions must acknowledge the same subjection to influences beyond their control, as that which attaches to the art whose province it is to deal with the living, thinking human being, and must submit all their hopes to contingencies, which they can neither foresee nor prevent.

The agriculturist, who, with a practical knowledge of his pursuit, has an intelligent acquaintance with the principles which influence it — with the control of climates and seasons, and with the nature of soils — has a much greater chance of success, than one who works in blindness and ignorance, and during a series of years his average returns will be much greater and more secure; but with the application of all his knowledge and skill, he cannot on any one year feel an assurance of success. Droughts may parch his fields, or floods drown them, frosts nip his fruits in the bud, or blight his grain in the ear.

The prudent and intelligent navigator, carefully studies the currents which sweep noiselessly through the ocean, and the laws which govern the winds blowing over its surface, and when his vessel is trusted to this vast machinery of nature, it is in no ignorance of the forces and powers to which it is subjected. The addition made of late years to the stores of nautical knowledge in all its branches, has not only shortened the time of distant voyages, but added to the total average security with which the ocean is traversed, and it is evident that the probabilities of a safe voyage will be greater for the vessel in proportion to the intelligence and professional acquirements of those having her in charge. But, the finest ship that ever floated, navigated with all the skill that man's intellect can display, may never reach her point — may be cast, a shattered wreck on the shore, or if she be brought into harbor, it may be dismounted, rigging, spars, and cargo all gone, and yet in this crippled condition, she may be the witness and trophy of more nautical science than if she had made her voyage unharmed.

Such considerations apply with yet greater force to the art of medicine, which deals, not only with nature and its physical law, but with the moral and intellectual constituent in man; and this wonderful combination of average and increasing success, with an ever existing uncertainty, is a beautiful exhibition of the harmonious laws by which the wisdom of Providence reconciles apparent contrariety. A general and progressive success is given to stimulate man to general and progressive exertion but were he to reach certainty where the laws of nature are concerned, he would be robbing the Deity of his prerogative, and become independent of his Maker; and hence all the arrangements and protections of science and philosophy are prostrated before the asserted omnipotence of the Deity, and yet man has no excuse to refrain from the acquisitions of knowledge.

It is an ignorance of, or want of reflection upon these principles, which forms the foundation for the prevalence of quackery. The pretender to medical science meets the popular expectation by promising infallible remedies for every disease. The quack, however, is not always an impostor. He partakes of the popular ignorance, and popular expectation, and promises infallibility because he believes infallibility to be a possibility; and ignorant of the laborious processes of scientific induction, believes that he can jump at the results of those processes, as certainly as those who reach them by the steps of learning.

The reasonable expectations of professional usefulness being thus lost sight of — the common sense means — those in which cause and effect bear an apparent relation, are set aside also, and to accomplish wonderful expectations, wonderful means are resorted to — means whose mode of action is as incomprehensible as their expected effects are inconsistent with the teachings of nature, and the designs of Providence.

For all this ignorance, misconception and error, a fearful retribution is visited upon the community. It pays the penalty of half its life, and consequently half its usefulness and happiness. With all the elements of health and long life in our country, statistics lead to the fearful conclusion that our average length of life is but little, if any more than half that enjoyed by overcrowded, overworked, vicious, and half-starved Europe. There, some care is taken that those to whom is entrusted the health and lives of the people shall be qualified for their duties by suitable education. Here, every ignorant or conceited pretender is permitted to assume the solemn responsibility of managing the health of his fellow-citizens, and their lives become the playthings of his blind folly and vain presumption.

That so terrible an evil as that which results from the existing popular notions of the art of healing, should permanently continue in an enlightened community or age is impossible. The dawn of a better day is seen in the fact that the Executive of the State of New York has made special mention of the subject of medical education. The following is taken from a message of the late governor of New York: "No subject more universally affects all classes, and all members of the community, than that of the public health. I therefore earnestly request your attention to the existing laws on the subject, and suggest their careful review and amendment, especially with a view to secure the benefit of the combined experience of scientific and learned men throughout the state, with respect to the origin, the causes, the progress and the treatment of all malignant or infectious diseases. It may also be well to consider whether the time has not arrived when the state is called upon to contribute its aid, more efficiently than it has hitherto done, to advance the cause of medical education. Every inhabitant of the state, at some time or other, feels the need of the physician, and is interested that he should be learned and skillful."

"Learned and skillful," yes! these are the requisites, but how are they to be attained? There are medical schools in the north, the south, the east, and the west, and every year sees these schools sending forth crowds of young men certified as being qualified, "learned and skillful" in the art of healing, and this after a term of study too short to acquire thoroughly any one of the sciences which in the aggregate make up the profession of medicine. Almost every country neighborhood sees some young man too sickly, too lazy, or perhaps too stupid to learn a mechanical pursuit, go off to study medicine, provided that, for two or three winters, he, or his friends can raise a few hundred dollars; and at the end of this time he comes back a qualified doctor, with a diploma in his pocket. Every one knows that he was entirely deficient in that preliminary education which is the key to professional knowledge. The people soon learn to feel as much respect for the spontaneous quack of the neighborhood, as for him of college growth, or perhaps the natural shrewdness and intelligence of the former give him an advantage.

Of course so long as the granting of diplomas is a mere trade, and medical schools are but shops for their sale, the multiplicity of these shops begets a competition which lowers the terms and standard so as to attract the greater number of customers, and those who should be the guardians of the profession of medicine, send forth such representatives of it, as lead to the erroneous popular opinions of the nature of the science itself.

No one may here oppose the popular objection that we would limit the facility for acquiring a knowledge of the profession of medicine to the few whose wealth would enable to attend expensive schools remote from their homes. Just the reverse; we would make the domain of medicine a true republic, and only ask of its members if they have the requisite knowledge; not, where they got it; — whether amid the halls, laboratories, and libraries of large cities, or, through the inspiration of genius, by the light of a pine torch in a forest cabin. Let every village have its medical school, if it may be thought expedient. Indeed it is a question whether, if medical education, or a knowledge of the principles of medicine were a part of general education, there would not be greater confidence in the profession, and more respect awarded to those who pursue it? An illiterate person might apply to a quackery juggler, or to any one less illiterate than himself, for information upon some abstruse point of chemistry, geology, minerolagy, or astronomy, but all having only the ordinary school knowledge of these sciences, would know that only those eminent for their learning would be likely to give the required information. The same thing is seen when regularly instructed members of the profession apply for information to those of the profession having greater skill and learning than themselves. Many gentlemen, particularly in the southern states, study medicine for the sake of mental occupation, with no intention of pursuing it as a business, and many who have been in the profession abandon it for other pursuits, yet these gentlemen, who are informed upon the nature of the profession, seek the best attainable medical advice for the relief of the ailments of themselves or their families. These facts lead very strongly to the inference that popular medical education would be fatal to the existence of quackery, and would leave the practice of medicine in the hands of those having the best natural and acquired gifts for its pursuit, and those so gifted would occupy a high and honored place in the confidence of an intelligent constituency.

Whilst then we make no objection to the multiplication of schools and teachers of medicine, we doubt very much the expediency and propriety of these schools and teachers having the interested power to grant diplomas — to certify to the merits of their own numerous offspring; and while this system endures, the public is justified in its contempt for diplomas. It is suggested that a better mode would be to compose an examining board in each state, in which all the schools might be represented, and thus examine each other's pupils, or all candidates presenting themselves; the faculty of the state might also be represented, and even the people, through their executive or a committee of their legislature.

If some such system as this were adopted, the results would show what teachers and schools were worthy of most confidence; the despotism of institutions which have grown powerful from adventitious circumstances would be broken down, and the people, having a part in the process of conferring medical degrees, would be more ready to oppose quackery by legislation.

In setting forth the influences which tend to degrade the profession of medicine from its true and high position, it would be a serious and disrespectful omission to say nothing of that of the public press, at once the exponent, and controlling power of public sentiment. If the stately essays and the dignified leaders of respectable papers are alone taken into consideration, the profession of medicine has nothing to complain of. These generally pay a formal tribute to scientific principles, institutions, and men. Their columns contain paragraphs for the instruction of the people, and cautions against humbug, deceit, and imposture; but turn to the page of advertisements, and, for the lure of an advertising fee, we find columns of absurd notices of quacking pills and potions, such palpable impostures as to have no influence with the educated and discriminating, but intended, and too successfully effecting the intention of deceiving the ignorant and unthinking. None but those whose professional avocations bring them into association with the humble and laboring classes, can imagine the amount of money which is robbed from these classes by such advertisements, particularly in the country districts; and the amount of disease and suffering caused by these ignorantly compounded, and ignorantly administered poisons is deplorable. Many of these notices are upon subjects which should never be obtruded upon the public eye, and convey licentious and obscene ideas into the bosom of families, and they propagate the vices for whose effects they pretend to offer a remedy. All this is certainly a great moral wrong, and it argues much against the moral sense of the community that the press, the assumed custodian of the public virtue, shall be guilty of this wrong, and yet claim to be respectable.

The list of various and contrary diseases which these nostrums pretend to cure, is alone sufficient evidence of their false pretension, and should be such to those who become the vehicle of imposing the falsehood upon the public.

Any of the ordinary and every day diseases of which people complain, may have their origin in a variety of morbid changes, each one requiring a different mode of treatment, and of course, no one means of cure being applicable to all. We will take "headache" as an illustration. This painful and distressing affection may have its cause within the contents of the skull, or in its external covering, and may arise from different affections of these parts. It may, as is most frequently the case, depend upon derangement of remote organs — the stomach, liver, or bowels. It may be a symptom of debility or of a too full and plethoric habit. The following list of causes which excite headache into action, is taken from the Cyclopœdia of Practical Medicine, and they are sufficiently varied to show that no remedy will reach all.

"1. Rheumatic affection of the pericranium. 2. Inflammation or a more chronic morbid condition of the pericranium. 3. Inflammation of the mucous lining of the frontal sinus. 4. Intense mental excitement. 5. Strong impressions on the external senses. 6. Excessive impetus of blood to the head. 7. Impeded return of blood from the head. 8. Congestion within the head. 9. Suppression of accustomed evacuations. 10. Inflammation of the brain or its membranes. 11. Tumors, or other morbid changes of structure within the head. 12. Morbid affections of the stomach;— as from over excitation or distention; from irritating ingesta; from imperfect digestion; the presence of bile in the stomach, &c. 13. Costiveness. 14. Narcotics. 15. Worms. 16. Diminished pressure of the atmosphere. 17. A heated, humid, or deteriorated atmosphere. 18. Sudden changes of temperature. 19. Exposure to a current of air, or to a cold wind, especially from the east." Headache has been chosen as an illustration, from the frequency of its occurrence, and not because it has a more varied origin than any other of the every day diseases for which quack remedies are offered.

It is not probable that those who are most exposed to the wrong of quack advertisements, will see these remarks, and hence it is the more incumbent upon all who may accord in the views now presented, to use their influence to prevent the spread of dangerous errors amongst those classes of the community, in which they may do the work of mischief, and be without the reach of correction.

Assailed as the profession of medicine is by popular misconception; misrepresented by unworthy members, and unsustained by legislative protection, it is thrown upon itself for protection, purification, and elevation, and these ends it is now endeavoring to accomplish by voluntary association. The "American Medical Association" or national congress of the profession, organized by members from the various medical literary institutions, from state and county societies, is using its influence to elevate the standard of medical education, and is concentrating the wisdom of the profession in this country upon the investigation of subjects of importance to the health and happiness of the community. It has promulgated, for the guidance and government of its members, a code of morals defining the duties of medical men to the community, and to each other, upon principles of courtesy, honor, and christianity, and preventing the evil results of local rivalries and jealousies. The county societies serve as tribunals or courts to secure the observance of the moral laws of the profession. They also indicate to the people what medical men are in good repute with their brethren, and under obligations for the conscientious performance of their duties. These local societies also collect from their vicinity, and from the members who form them, those facts of interest to the public weal, which would otherwise be lost, but being contributed to the general store, form a vast amount of valuable information; for, it is a mistake, and one fruitful of evil to the community, to regard the medical profession as limited in its duties to the relief of the sick who may come under the charge of individual members of the profession. It has a far more extended mission than this. To remove the general sources of disease — to prevent sickness and suffering — to ascertain the physical and moral sources of human depravity — and to indicate the means of their removal, are among the high objects of professional organization; and most nobly has the profession come up to its work. Notwithstanding the vulgar, illiberal, and ignorant sneers of those who charge upon medical men a wish for the increase of disease, their profession has been found the most active in the promotion of measures of general health and sanitary reform; although in its labors it has had to contend with popular prejudice, legislative indifference, and opposing pecuniary interests.

The subjects bearing upon general and individual health, to which professional attention has been of late actively directed both in Europe and America, are, the water supply and sewerage of towns and villages; the drainage of the soil; the construction, arrangement, and ventilation of dwellings; the investigation of cholera and other epidemics; the establishment of public baths and wash houses; the registration of marriages, births, and deaths; and the temperance reform. The efforts of the profession to arrest the evils of quackery are correspondent to its whole sanitary action, although, unfortunately, they are attributed to interested motives, whereas, in truth, the evidence goes to show, that the more quackery prevails, the more is the employment for scientific medicine, and the "Family Medicine," whether a book or a pill, is a fertile source of fees to the family physician.

The organization of the profession in county, state, and national associations, has been efficient, among other matters, in calling the attention of legislators to the necessity of the law for the registration of births, marriages, and deaths. The following remarks upon this subject, are taken from the "Report of the Committee of the House of Representatives of Pennsylvania."

"The Registry Law would teach the laws of human life developed by the natural constitution of our bodies as they usually exist under the influences that surround them, and how far they may be favorably modified and improved. This can only be done by an accurate knowledge of the facts that are daily occurring among us. These matters are important to the physician to aid him in curing the sick, but far more important to the people to aid them in learning how to live without being sick." It is estimated that the annual loss in England and Wales alone by preventable disease is greater than the loss of the allied armies at the battle of Waterloo. In the county of Lancaster, eleven thousand adults die annually of removable epidemics, and it is further estimated that the annual pecuniary loss of the United Kingdom by preventable disease, is one hundred millions of dollars. A committee of Parliament report upon a registration law, that, "It involved matter of great public and national interest as well as individual satisfaction; and rights and claims to property; that great trouble, vast expense, utter uncertainty, capricious changes, and local and general evils exist, while no means are supplied to obtain the information other countries possess and greatly value, as to the state of disease, the operation of moral and physical causes on the health of the people, the progress of the population and other matters, on which accurate knowledge can scarcely be too highly appreciated or too intensely pursued." A consequent bill introduced by Lord John Russell became a law in 1837. "The medical profession, with all that science and philanthrophy that everywhere distinguishes them, have wrought upon these rich and abundant results of a varied registration, and elicited great truths. By sharp scrutiny, close and laborious comparison, they have established the comparative health of localities, and with an industry not less active, having discovered the cause of disease, have pointed out the means of its removal." The following eloquent remarks are from an article upon "Sanitary Reform" in the British and Foreign Medico-Chirurgical Review:

"The quarterly reports of the Registrar General are among the most interesting and instructive documents of the day. They are to us what, in an inferior degree, the Saxon Chronicles were to the eleventh and twelfth centuries. They engrave, in brief but expressive phrases, the national vicissitudes, prosperities, trials and calamities. With these faithful and unerring indices, marriages and deaths, the Registrar General measures the robustness of natural vigor, or probes the depth of national suffering. Backed by those ranks of expressive figures, which permit no exaggeration, and are susceptible of no fallacy, he presents to us a true picture of our country and nation. No false rhetoric, or untrue coloring is suffered to mar the truth of the hard and simple outlines. No political creed conceals the facts, or perverts their meaning. No unjust law orders the distortion of half the truth, by the concealment of the other half. These reports are, indeed, something more than history; they are the judgment of the time upon itself, and untinctured as they are by party spirit, and unswayed by personal considerations, those judgments are as true and faithful as those of future times can be. It is no objection to the value of these records, to say they chronicle with greater minuteness and accuracy the national ills and chastisements, than the national happiness and success. The most dreary and painful side of human existence, is certainly most largely presented to us. The shadow of imperfection and decay tinges all things with its melancholy hues. Our path is rather through the gloomy valley, and under the shade of cypress, than on the invigorating mountain side, resplendent with the light of Heaven. But this seems to be the necessary result of all true histories of the social condition of the people. That which is strongest and most permanent presses aside that which is less vigorous and enduring. Happiness and comfort escape the chronicles; gaunt features of misery and distress are ever before him. The happy hours of a nation's, as of an individual's life, are as the dowdy ripples which the advancing tide mashes into smoothness; the hours of sorrow and of trouble are like those ripples fossilized into stone."

The enlarged sphere of duty pertaining to the profession of medicine, can only be properly met by professional organization. The people have their most solemn interests concerned in sustaining the organization, and have much reason to suspect those who affect to be independent of it. Medical men who voluntarily refrain from the work, are either behind the age, ignorant of their duties and of what the profession is doing, or else are seeking to hide sinister designs and selfish purposes under an affectation of individual independence, just as all do who profess to be independent of the general laws of society. It is an easy mode of getting rid of wholesome obligation and restraint, by assuming to be entirely independent of it, and the people who cheer on such lawless spirits, must not complain if they find themselves the victims of lawlessness. An irregular practitioner in one of the western counties of Pennsylvania, seeking to trap this popular sympathy, advertises himself, in the most triumphant manner, as *independent of the*

*county medical society*: — a society to which he is of course entirely ineligible. Men may affect this independence who have an interest in shunning the light of professional investigation, and may claim the privilege of darkness as an independent right; but it cannot be awarded them; the interests of society forbid it. If the professional man is weak, he owes it to those committed to his professional charge to have the aid and council of his professional brethren, and if he is strong, he owes a portion of his strength to the profession and the good of society. The egotism of individual vanity judgment, and interest, must be under subjection to the "Higher Law" of christian, professional, and general organization, or christian, professional and general communities, have no security for the conduct of their members.

Those who take a correct view of the various difficulties we have suggested as connected with the practice of medicine will see that it is not a profession whose paths are through smooth and flowery walks. But it may be as well to offer a few considerations for the use of those who are desirous of entering that profession, and, in doing so, our desire is not to diminish the number of "doctors," but to increase the number of those who are qualified to perform the obligations they take upon themselves. It is a maxim with political economists, that cheapening an article increases its consumption, and, in the aggregate, invites a larger expenditure of money upon that article. The maxim is perfectly true in regard to cheap doctoring: it costs the people a vast amount of money, and all that is received in return is a worthless and injurious article. It may well be questioned whether the same degree of benefit accrues to the people by cheapening their facilities for medication, as arises from the lessened prices of silk and cotton fabrics. A case, unhappily, of every day occurrence, will illustrate this position. An individual is affected with some chronic, permanent, and incurable ailment, and yet it may not be one seriously affecting his life and usefulness: Upon consulting some medical man well and thoroughly skilled in the laws and nature of disease, after, perhaps, not more than fifteen minutes conference, the patient is told that the use of medicines can be of no service to him, and will only be a fruitless expenditure of his money, his time, and his constitution. If charged for this advice five, ten, or twenty dollars, he may regard it as an outrageous robbery, and yet for want of this learned and honest counsel, he may go from doctor to doctor, and from quackery to quackery, paying but fifty cents here and a dollar there, not for advice, but for medicine, until he has expended hundreds of dollars, weeks, months, and years of time, and all the vigor of constitution which was necessary to sustain him under the burden of his disease. Hence, fewer and more competent medical advisers would be more to the profit and happiness of the people, and service will be done them if a due reflection upon the difficulties of the profession shall deter any of the crowds now rushing to the medical schools for their purpose.

The entire difficulties of the study of medicine are rarely understood by those who commence it; but it may be appreciated when it is considered that the mental faculties and the physical powers are to be given to the acquisition of five or six different sciences, and to the languages of those sciences. The powers of memory, of observation, of judgment are to be assiduously cultivated, and with no end to the labor. Those who have studied a language know the time and labor it costs, and those who have not the mental discipline as well as the facilities arising from the acquisition of a language, are unfit for the studies of medicine; and yet the acquisition of language is easy and agreeable, relatively to that of medical science. The medical student may and should have his healthful exercise; but he has no right to amusements or relaxations which distract his thoughts or lessen their vigor. He must have that devotion to philosophy and love of truth which will make the most repulsive investigations agreeable, and keep him days and nights, for much of his life, in damp dissecting rooms, in hospitals, and pestilent dead houses. The severe discipline of mathematics must have trained his reasoning powers, and his eye and hand have been taught by skill in drawing. If his physical constitution be not good, he will sink beneath the burden of study and exposure, and add to the number of those, who are conducted to the grave by consumption and fever before their studies are completed.

The granting of the college certificate, or diploma, is very far from ending the student's labors, it only opens to him another course of education and more pressing obligations to pursue it. If the diploma has been his object, he is unfit for a profession which imposes upon its members the most sacred obligations to preserve life, and to relieve pain, suffering and sorrow. If his object is merely to make money — to acquire fortune and honor, or to live at ease, he is as unfit for the profession as the profession is for him. Statistics go to show that among occupations that of medicine is the shortest lived, and the least successful in the accumulation of wealth. He must look for his reward to his own breast, in the consciousness of being able to do his duty, and in having done it; even though misjudged and censured by those whom he has benefited, and who are competent to form any idea of his capabilities or of the long and anxious labors by which they have been reached. He belongs to a profession which gives him frequent opportunities of practising upon the command: "Do good to those who despitefully use you." He must give up all command of his time night or day, and be prepared any and every moment for the most harrassing emergencies. Even when his active duties may not call him from his home or bed, he must expect to pass anxious hours and sleepless nights from the responsibility of intricate cases, and the consciousness that the lives and happiness of others are dependent upon his skill and judgment, and yet, after all this, he may find every one, from the shoemaker's bench to the pulpit, advancing the most positive and dogmatic opinions upon medical subjects in opposition to his own, and recklessly prescribing for patients which are to him such a source of mental anxiety;

"As fools rush in
Where angels fear to tread."

After large contributions of gratuitous labor to the poor, he must be content to see his services valued, by those able to pay him, by the standard of day labor, and may consider himself fortunate if he is not, when he has done his best, dragged before an ignorant and hostile jury, vilified and traduced by hireling lawyers, and robbed of his means and reputation by the testimony of unprincipled and rival quacks.

Such are the contingencies of the life of the skillful and conscientious physician; still more unhappy is the position of him who has undertaken obligations which he is naturally incompetent to meet, or for which he has not fitted himself. When such a one as this enters the darkened chamber of disease, and feels the anxious and hopeful gaze of relatives penetrating his soul, and yet is at a loss for resources to meet the demands made upon him; is bewildered, not by the intricacy of the case, but by his conscious inability to meet it, and fears that the trust reposed in him is a false trust, which must close in disappointment, sorrow, and death, when proper medical qualification would be able to give cheerful encouragement, and to change the gloomy scene to one of happiness and joy: then, if he has any feeling of man, he reproaches his misplaced position, and remembers with bitter agony the hours wasted in selfish enjoyment, which should have been given to the solemn duties of his calling.

Better for the peace and happiness of such a man had he sought any trade or occupation, however humble and laborious, so that it was but honest and suitable to his character and abilities.

We have endeavored in this paper to draw the distinction between the true nature of the profession of medicine, and the popular view taken of it, to point out the causes of error and to suggest certain remedies, which, if efficient for the purpose, will benefit the public by restoring the profession to its proper place, and making its representatives equal to the obligations they have taken upon themselves. Whilst health, life, and morals are matters of general import, it is the duty of the educated classes, the pulpit, and the press, to aid the efforts now making by the medical profession to confer upon the people the benefits of true science, and to protect them from ignorance and imposture. The magnitude of the existing evil needs only to be known, to call for the energetic action of every conscientious member of society.

